# Risk Taking in Hospitalized Patients with Acute and Severe Traumatic Brain Injury

**DOI:** 10.1371/journal.pone.0083598

**Published:** 2013-12-26

**Authors:** Shirley Fecteau, Jean Levasseur-Moreau, Alberto García-Molina, Hatiche Kumru, Raúl Pelayo Vergara, Monste Bernabeu, Teresa Roig, Alvaro Pascual-Leone, José Maria Tormos

**Affiliations:** 1 Centre Interdisciplinaire de Recherche en Réadaptation et Intégration Sociale, Centre de Recherche Universitaire en Santé Mentale de Quebec, Medical School, Laval University, Quebec city, Quebec, Canada; 2 Berenson-Allen Center for Noninvasive Brain Stimulation, Beth Israel Deaconess Medical Center, Harvard Medical School, Boston, Massachusetts, United States of America; 3 Institut Guttmann, Institut Universitari de Neurorehabilitació adscrit a la UAB, Badalona, Barcelona, Spain; Universitat Autònoma de Barcelona, Bellaterra (Cerdanyola del Vallès), Spain; Fundació Institut d’Investigació en Ciències de la Salut Germans Trias i Pujol, Badalona, Barcelona, Spain; University of Pittsburgh, United States of America

## Abstract

Rehabilitation can improve cognitive deficits observed in patients with traumatic brain injury (TBI). However, despite rehabilitation, the ability of making a choice often remains impaired. Risk taking is a daily activity involving numerous cognitive processes subserved by a complex neural network. In this work we investigated risk taking using the Balloon Analogue Risk Task (BART) in patients with acute TBI and healthy controls. We hypothesized that individuals with TBI will take less risk at the BART as compared to healthy individuals. We also predicted that within the TBI group factors such as the number of days since the injury, severity of the injury, and sites of the lesion will play a role in risk taking as assessed with the BART. Main findings revealed that participants with TBI displayed abnormally cautious risk taking at the BART as compared to healthy subjects. Moreover, healthy individuals showed increased risk taking throughout the task which is in line with previous work. However, individuals with TBI did not show this increased risk taking during the task. We also investigated the influence of three patients’ characteristics on their performance at the BART: *Number of days post injury, Severity of the head injury*, and *Status of the frontal lobe*. Results indicate that performance at the BART was influenced by the number of days post injury and the status of the frontal lobe, but not by the severity of the head injury. Reported findings are encouraging for risk taking seems to naturally improve with time postinjury. They support the need of conducting longitudinal prospective studies to ultimately identify impaired and intact cognitive skills that should be trained postinjury.

## Introduction

Traumatic brain injury (TBI) is an important cause of disability among adults, resulting in tremendous human and financial cost [1], [2]. A TBI is caused by external forces, such as a blow, jolt or penetration to the head temporarily or permanently disrupting brain functions [3]. Thanks to advanced medical care, the survival rate of patients with TBI continues to increase [4]–[6]. However, patients can experience lifelong impairments across virtually all domains of functioning, including physical, cognitive, emotional, behavioral, and social areas [7]. Cognitive and behavioral deficits seem to be especially deleterious impacting overall functional outcomes and quality of life of patients and their families [8]–[15]. The extent of these deficits following TBI is broad and includes information processing [16], attention [17], memory [18], executive function [19], computation, and discrimination of probabilities [20]. They can occur singly or in combination, can change in severity over time, and often remain greatly impaired despite rehabilitation [10], [21]–[23]. These processes need to be integrated when assessing the risk to take or not relative to making a choice and, indeed, often remains impaired long after the injury and can critically hinders social autonomy [24]. It thus seems important to carefully identify impaired and intact processes involved in risk taking and characterize factors that may be associated with them (e.g., time course, severity of the injury). Such further knowledge may contribute to eventually develop effective and individualized rehabilitation program to improve risk taking in individuals with TBI. We adopt here the definition of risk taking proposed by Leigh [25], that is “behaviors that involve some potential for danger or harm while also providing an opportunity to obtain some form of reward”.

In the present study we investigated risk taking behaviors in TBI patients using the Balloon Analogue Risk Task (BART). Our general hypothesis was that subjects with TBI will display risk aversive response style at the BART as compared to healthy controls. This prediction was based on previous work [26]–[28] reporting impaired risk taking and decision-making in TBI patients compared to controls, using however more complex tasks (e.g., the Iowa Gambling Task) than the BART task. The BART is a behavioral measure of risk taking with real-world convergence. Specifically, it has been correlated with occurrence of real-world risk behaviors [29]–[31], such as substance use [32], risky sexual behaviors [33], and delinquent behaviors [29–31,34], as well as self-report measures of risk-related constructs such as sensation seeking, impulsivity, and deficiencies in behavioral constraints [29]. In the context of the present study involving patients with TBI, the BART has the advantage of not involving working memory and calculation *per se*. This is particularly important when studying performance of patients with acute TBI as memory and attention are often impaired [35] and could be confound factors in the study of risk taking. Obviously in real world situations, numerous cognitive processes are involved when taking risk [36], [37]. However, the literature on risk taking behaviors in acute TBI patients is still scarce and there is a need to gather a fine characterization of specific deficits contributing to impaired risk taking behaviors in this population. Here we chose to focus on aspects of risk taking that do not involve memory or calculation. Aspects of risk taking studied at the BART involve choices made in the context of increasing risk in ambiguous situations [29]. Ambiguity can be viewed as any situation in which the likelihood of one or more of the payoffs occurring is not fully specified [38], [39]. The BART meets this definition of an ambiguous decision in that one of the choices (to make another balloon pump) has unknown probabilities. At each decision point, the participant has to make a choice between a chance for an incremental gain or potentially larger losses with unknown probabilities versus a 100% certainty of no loss but no additional gain.

We also investigated whether performance at the BART in individuals with TBI differed according to the *Number of days post injury*, the *Severity of the head injury*, and the *Frontal lobe status* (described below).

## Methods

### Participants

Individuals with acute TBI and healthy adults took part of the study. The TBI group was composed of 30 in-patient subjects at the Institut Universitari de Neurorehabilitació Guttmann-UAB (5 women) with an average age of 31 years old (range of 16 to 58 years). Severity of their initial injury was diagnosed using the Glasgow coma scale (GCS) with mild (N = 3; GCS ≥ 13), moderate (N = 6; GCS 9–12) or severe TBI (N = 21; GCS ≤ 13). The average number of days between their injury and their participation at this experiment was 129 days (range of 44 to 288 days). The healthy group consisted of 8 adults (2 women) with an average age of 28 years old (range of 19 to 37 years). Exclusion criteria for the healthy subjects were aged under 18 years old and history of any neurological or psychiatric disorders. Participants or their legal guardian gave informed written consent prior to entering the study, which was approved by the Institut Universitari de Neurorehabilitació Guttmann-UAB ethics committee (Barcelona, Spain). Please refer to Table 1 for details.

**Table 1:** Characteristics of the subjects.

Subjects	Gender	Age	N of days since the injury	Glasgow score
**P1**	M	27	44	9
**P2**	M	35	44	3
**P3**	M	28	45	0
**P4**	M	29	45	5
**P5**	F	27	52	7
**P6**	M	25	54	5
**P7**	F	58	55	13
**P8**	F	26	68	5
**P9**	M	36	71	13
**P10**	M	23	87	11
**P11**	M	24	88	6
**P12**	M	31	92	0
**P13**	M	24	97	6
**P14**	M	24	99	9
**P15**	M	36	103	3
**P16**	M	57	104	15
**P17**	F	30	107	9
**P18**	M	38	133	0
**P19**	M	19	154	5
**P20**	M	37	155	3
**P21**	M	32	156	7
**P22**	M	29	170	12
**P23**	M	25	171	4
**P24**	M	34	176	5
**P25**	F	16	193	4
**P26**	M	31	211	4
**P27**	M	26	241	4
**P28**	M	38	282	3
**P29**	M	50	286	0
**P30**	M	22	288	9
**H1**	F	29	-	-
**H2**	M	29	-	-
**H3**	M	20	-	-
**H4**	M	28	-	-
**H5**	M	19	-	-
**H6**	M	33	-	-
**H7**	M	37	-	-
**H8**	F	31	-	-

### Balloon Analogue Risk Task (BART)

**Figure 1:**
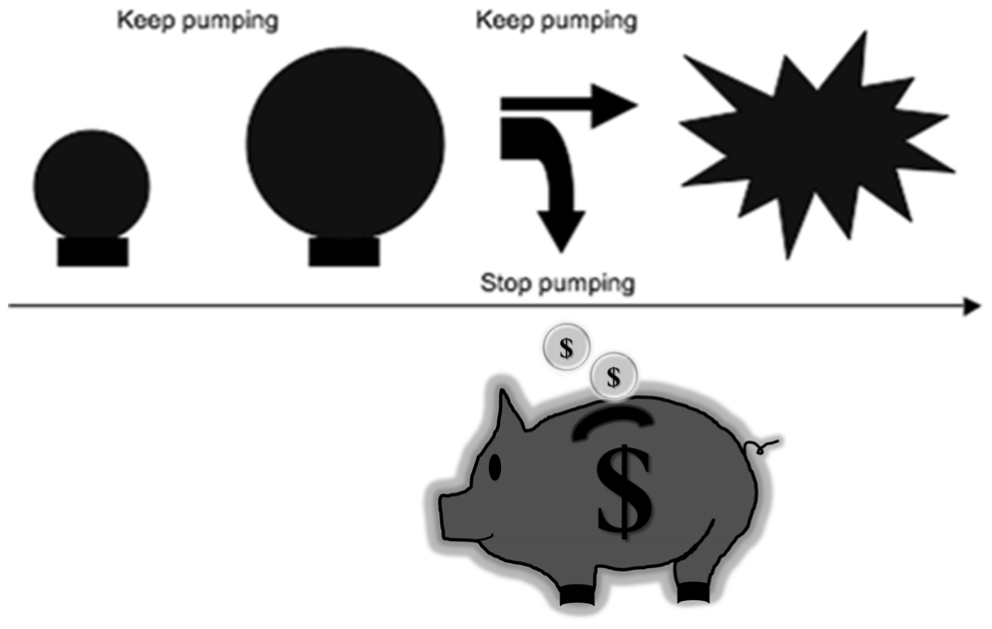
Schematic representation of the BART experiment.

In the BART, participants have to make a choice in a context of increasing risk. They are invited to inflate a computerized balloon by pushing a ‘pump’ (see Figure 1). The balloon can explode at any moment. Participants have to decide after each pump whether to keep pumping and risk explosion of the balloon, or to stop. Participants accumulate money in a temporary bank with each pump (5 cents for each pump). When the participant decides to stop pumping, the accumulated money is transferred to a permanent bank. However, if the balloon explodes, all of the money accumulated in the temporary bank is lost. Therefore, the probability of losing the money, as well as the potential lost (i.e., the amount of money) increases with each pump. Each balloon has a different explosion point. There are a total of thirty trials (balloons). The BART was conducted in an experimental room equipped with a PC computer. Instructions for the BART task were written so that all participants received the same instructions, and participants were invited to ask any question they may have after reading the instructions. They were given no precise information about the probability of explosion or the total amount of money acquired from previous participants. Participants were told that the subject with the highest amount of money would receive a gift certificate.

### Patients’ characteristics

In order to further explore risk taking behaviors in TBI patients, we tested whether performance at the BART was related to any one of the three following patients’ characteristics.

**1. Number of days post injury.** We tested whether the number of days between patients’ injury and participation at the BART experiment was related to the BART performance. Although the general ability of risk taking seems to remain impaired in TBI patients, some cognitive functions involved in risk taking, such as attention and information processing, may improve at specific periods post-injury with usual rehabilitation therapy [21], [22], [40]. It thus seems important to identify whether risk taking remains impaired regardless of the number of days since the injury or whether it indicates a natural recovery or improvement. Based on studies suggesting cognitive improvement with functions associated with risk taking [21], [22], we hypothesized that within the TBI group, performance at the BART will be impaired (that is they will be risk averse), especially for patients with the smallest number of days since injury.

**2. Severity of the head injury.** The second patients’ characteristic we explored was the severity of the head injury. This was measured with the Glasgow Coma Scale (GCS). The GCS is one of the most common methods to characterize severity of head injuries [41]. This scale relies on observation of orientation, movement and verbal abilities of the patient and yields from mild, moderate to severe classification on a continuous scale. We used GCS scores to test whether severity of head injuries were related to the performance at the studied risk taking task in our TBI patients. We hypothesized that within the TBI group, patients with more severe head injury (i.e., lower GCS score) will display lower performance at the BART (i.e., risk averse behaviors).

**3. Frontal lobe status.** The third patients’ characteristic we studied was the status of their frontal lobe, whether a lesion was reported or not on their medical records based on their CT or MRI scans. Of note, analyses of CT and MRI scans (e.g., lesion size) and inter-rater reliability tests were not performed. Lesion work and neuroimaging studies in healthy subjects have repeatedly associated risk taking and frontal lobes [36], [42]–[44]. Adults with ventromedial/orbitofrontal lesions appear to engage in immediate, higher rewards and seem insensitive to potential future losses [45]. There is still no clear consensus in regards to the frontal hemispheric contribution on riskier behaviors and impaired decision-making skills, but greater deficits have been found with lesion in the right frontal lobe [43], [44]. Based on this literature, we thus tested whether patients with damaged frontal lobe (N = 14) and those with spared frontal lobe (N = 16) performed differently at the risk taking task. We hypothesized that within the TBI group, patients with frontal lesions in the right hemisphere will take more risk than patients with no frontal lesion.

### Data analysis

The main outcome measure was the averaged adjusted number of pumps (i.e., number of pumps for balloons that did not explode), which has been advocated as the preferred dependent measure for the BART because it avoids the constraints on individual differences that occur on trials with explosions (for which there is a fixed limit of potential pumps; [29,34]). We also calculated the time course of performance on the adjusted number of pumps (i.e., 10 first balloons; 10 second balloons; 10 last balloons) because there is typically an increase of risk taking as the task goes (i.e., increased number of pumps). We also calculated the total of money earned (although this measure is inherently linked to the number of pumps) because the goal pursued by the subjects in this task is to earn as much money as possible. Finally, we calculated the averaged number of explosions. When testing for differences between the TBI and healthy groups, we used analysis of variance (ANOVA). We subsequently assessed homogeneity using the Mauchly's test and we applied the Greenhouse-Geisser correction when appropriate. Results with a *p* value ≤ 0.05 were considered significant for all statistical analyses. Statistical analyses were performed using SPSS software (version 21.0, SPSS Inc., Chicago). There was no difference between the TBI and control groups for the age (*t* (36)  = 0.812; *p* = 0.422) or gender (*X^2^* (1)  = 0.292; *p* = 0.589).

## Results

### Performance at the BART in TBI patients and healthy controls

**Figure 2:**
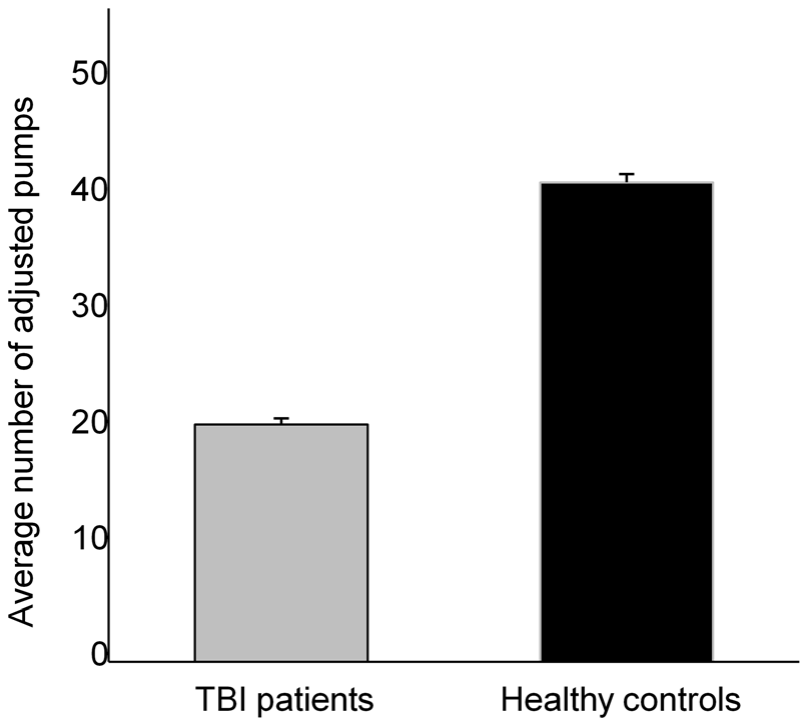
Graphic display of the average number of adjusted pumps (the total pumps of the balloons that did not explode) for each group. Error bars indicate SEM.

Examination of the BART data indicates a group difference on the average number of pumps on balloons that did not explode (AVOVA; *F*(_1,36_)  = 13.024; *p* = 0.001). As shown in Figure 2, the average number of pumps was smaller for the TBI group than the healthy group (20 and 40.4 pumps, respectively).

**Figure 3:**
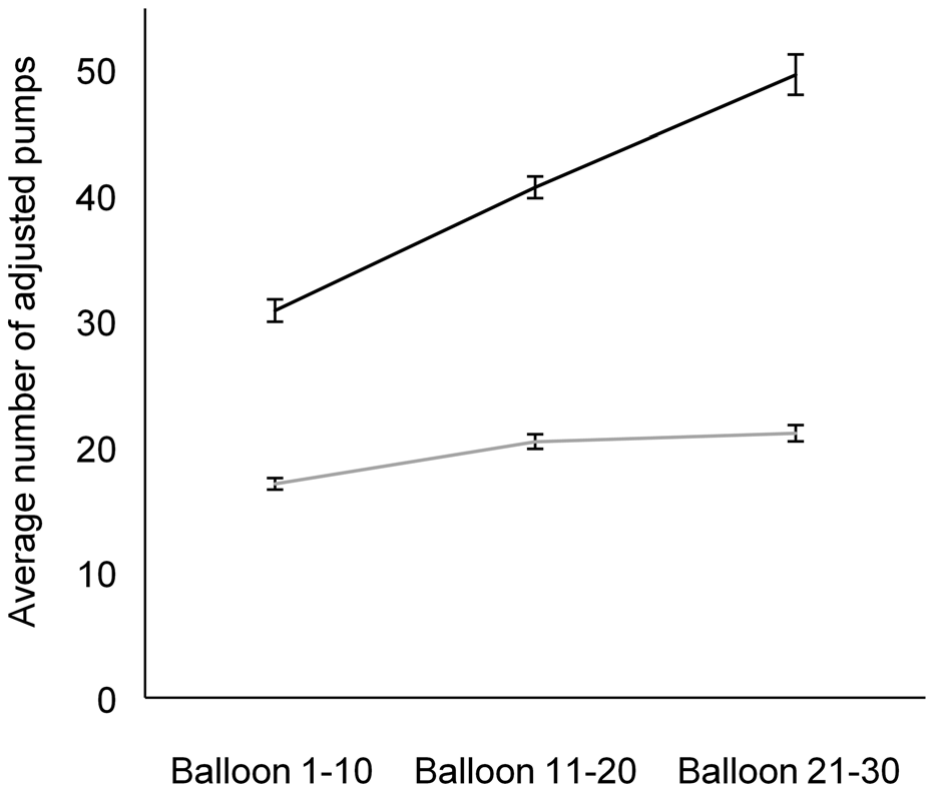
Graphic display of the average number of adjusted pumps for each group and time period (the first set of 10 balloons, the second set of 10 balloons, and the last set of 10 balloons). Black line represents the healthy group and the grey line represents the TBI group. Error bars indicate SEM.

For the time course at the BART (i.e., the average number of pumps on balloons that did not explode for the first 10 balloons, the second 10 balloons, and the last 10 balloons), there was an effect of group (repeated measures ANOVA with group and time as variables; *F*(_1,36_)  = 13.804; *p* = 0.001), an effect of time (*F*(_1.4,48.9_)  = 11.119; *p* = 0.001), and a significant interaction group by time (*F*(_1.4,48.9_)  = 4.641; *p* = 0.026). Post-hoc analysis (using Sidàk-Holm correction) revealed that groups significantly differed when comparing the first and second balloons (*p* = 0.038), the second and last balloons (*p* = 0.020) and when comparing the first and last balloons (*p* = 0.006). Control participants showed a substantial increase in risk taking with time. As illustrated in Figure 3, healthy controls pumped more toward the end as compared as the beginning of the experiment, whereas participants with TBI did not show a significant increase with time. We also ran the full model with a repeated measures ANOVA with these three groups (i.e., TBI with right hemisphere lesion, TBI with spared right hemisphere, and healthy controls) and time. We found an effect of group (AVOVA; *F*(_2,35_)  = 10.499; *p* = 0.0001), an effect of time *F*(_1.4,49.3_)  = 11.099; *p* = 0.0001), and an interaction of group by time *F*(_2.8,49.3_)  = 4.811; *p* = 0.006). Post-hoc analysis (Sidàk-Holm method) revealed a group difference between the TBI subjects with no right hemispheric lesion and the healthy subjects (p = 0.0001). These two groups also performed differently between the first and the second sets of 10 balloons (p = 0.006) and between the second and third sets of 10 balloons (p = 0.026). However, these groups did not significantly differed between the second and the third sets of 10 second balloons (p = 0.051).

There was also a group difference for the total amount of money earned (ANOVA; *F*(_1,37_)  = 10.541; *p* = 0.003). The TBI group earned a smaller amount of money than the healthy group ($22 and $39 respectively). There was also a group difference on the averaged number of explosions (ANOVA; *F*(_1,36_)  = 18.246; *p*<0.0001). The averaged numbers of explosions were 4.2 and 10.5 in the TBI group and healthy group, respectively.

### Performance at the BART and patients’ characteristics

**Figure 4:**
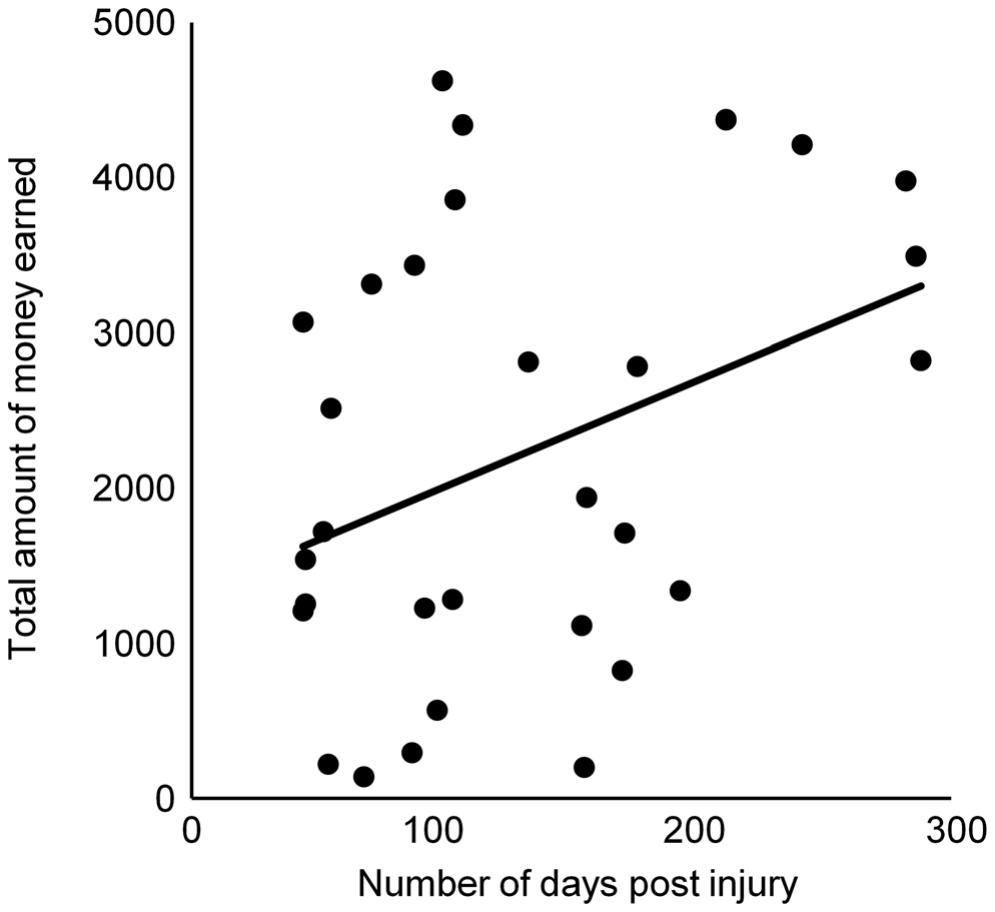
Graphic display of the mean amount of money for the TBI group according to the number of days since their injury.

**1. Number of days post injury.** The correlation between the main BART outcome and the number of days post injury (i.e., between the injury and the BART performance) was not significant (Pearson correlation; *r* = 0.330; 2-tailed value: *p* = 0.075). For the time course, the number of days post injury did not significantly correlate with performance in the first 10 balloons (*r* = 0.264; 2-tailed value: *p* = 0.158), the second 10 balloons (*r* = 0.335; 2-tailed value: *p* = 0.070), or the last 10 balloons (*r* = 0.333; 2-tailed value: *p* = 0.072). There was however a significant positive correlation between the number of days post injury and the total of money earned (*r* = 0.366; 2-tailed value: *p* = 0.047). This is illustrated in Figure 4.

**2. Severity of the head injury.** There was no significant correlation between the main BART outcome measure and GCS data (Pearson correlation; *r* = 0.111; 2-tailed value: *p* = 0.559). The GCS data was not correlated with the number of pumps on the first 10 balloons (*r* = 0.109; 2-tailed value: *p* = 0.565), the second 10 balloons (*r* = 0.184; 2-tailed value: *p* = 0.330), or the last 10 balloons (*r* = 0.240; 2-tailed value: *p* = 0.202). There was no correlation between the amount of money earned and the GCS data (*r* = 0.116; 2-tailed value: *p* = 0.542).

**Figure 5:**
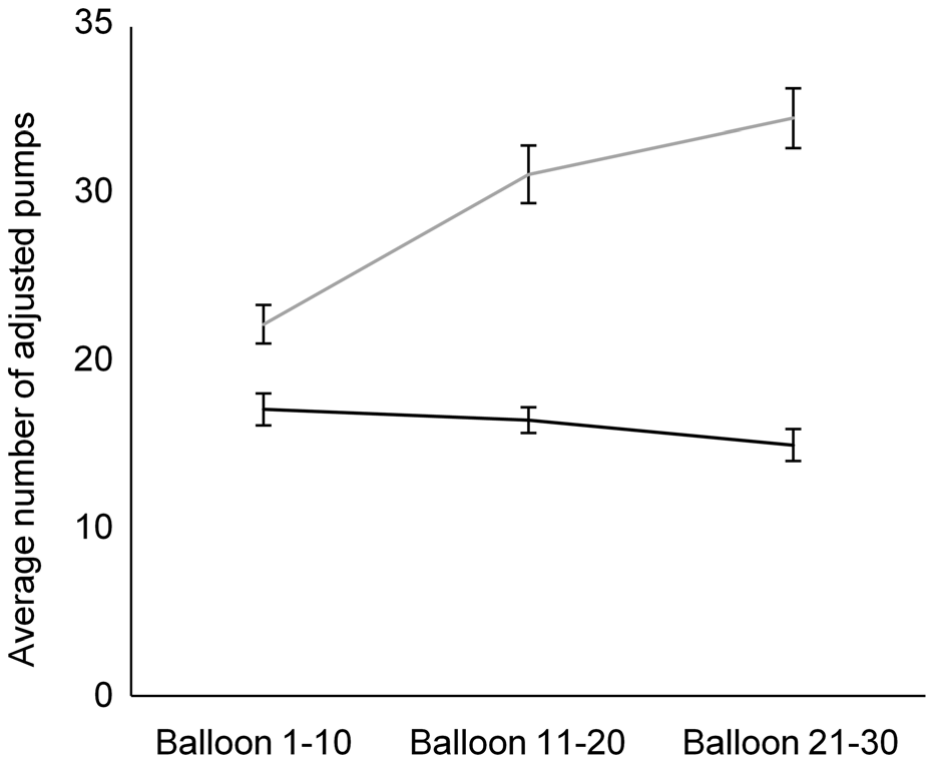
Graphic display of the average number of adjusted pumps for TBI patients according to whether or not they display a frontal lesion in the right hemisphere and time period (the first set of 10 balloons, the second set of 10 balloons, and the last set of 10 balloons). Black line represents patients with no frontal lesion in the right hemisphere and the grey line represents patients with frontal lesion in the right hemisphere. Error bars indicate SEM.

**3. Frontal lobe status.** There was no group difference when comparing patients with a frontal lesion in the right hemisphere (N = 14) to those with no frontal lesion in the right hemisphere (N = 16) on the main BART outcome measure (the average adjusted number of pumps; ANOVA; *F*(_1,28_)  = 2.563; *p* = 0.121). There was no group difference for the first 10 balloons (*F*(_1,28_)  = 0.770; *p* = 0.388). However there were significant group differences for the second 10 balloons (*F*(_1,28_)  = 4.568; *p* = 0.041) and the last 10 balloons (*F*(_1,28_)  = 6.897; *p* = 0.014). This is illustrated in Figure 5. There was no group difference for the total amount of money earned (*F*(_1,28_)  = 2.905; *p* = 0.099). We also examined for a possible link between performance at the BART and the presence or absence of a lesion in the left frontal lobe. None of the measures reached significance (ANOVA; main BART outcome (*F*(_1,28_) = 0.012; *p* = 0.915); first 10 balloons (*F*(_1,28_)  = 0.003; *p* = 0.954); second 10 balloons (*F*(_1,28_)  = 0.010; *p* = 0.920); last 10 balloons (*F*(_1,28_)  = 0.049; *p* = 0.826); money earned (*F*(_1,28_)  = 0.033; *p* = 0.857).

## Discussion

In the present work we investigated risk taking in patients with acute TBI and healthy controls. The main findings revealed that TBI patients displayed impaired risk taking. Indeed, they showed abnormally risk averse behaviors at the BART paradigm as compared to healthy controls. Moreover, healthy controls displayed an increasing risk taking throughout the task. This sort of learning curve throughout pumping the thirty balloons has been reported in previous studies [31], [32]. Subjects take more risk with the last set of ten balloons as compared to the second and first sets of ten balloons. This increasing risk was not found in the TBI group. Patients barely increased their risk taking throughout the task. This result differs from that of Chiu and colleagues [46]. These authors investigated the neural correlates of risk taking at the BART in TBI patients using functional magnetic resonance imaging. TBI patients and healthy subjects differed in terms of patterns of brain activations when performing the BART. However, behavioural responses at the BART (i.e., risk taking) was not significantly different between the two groups. As the authors mentioned, this negative behavioural finding may be explained by their small sample size (11 TBI patients and 8 healthy subjects).

We further investigated risk taking at the BART in TBI patients according to three characteristics. These variables were chosen in order to identify whether characteristics of the injury influenced risk taking. These were the *Number of days post injury, Severity of the head injury*, and *Frontal lobe status.*

**Effects of number of days post injury on risk taking in TBI patients.** There was a correlation between the number of days post injury and the amount of money earned at the BART. The smallest number of days post injury was related with the smallest amount of money earned. This effect was modest, but it suggests a natural improvement in risk taking soon after the injury, within the period of 44 to 288 days post-injury. This is in line with natural improvement of cognitive performance (e.g., executive functions, memory, processing speed, language abilities, constructional skills) that has been previously observed during the first year postinjury [47], with greater improvement during the first 5-6 months [48]. Of note, although risk taking seems to improve with time, it remained greatly impaired as compared to healthy subjects.

**Effects of severity of the head injury on risk taking in TBI patients.** Previous work indicates that cognitive deficits such as executive dysfunction are related to severity of TBI [47], [49], [50]. This was not observed here. Of note, our sample size was limited (N = 30) and the number of patients according to the severity of the TBI was unequal (three patients with mild TBI, six with moderate TBI, and twenty-one with severe TBI). This may explain the lack of group difference based on severity of the injury. Also, group difference based on the severity of the injury on cognitive skills has mainly been reported in chronic patients [47], [49]–[51]. It is possible that during the acute phase (as tested here), severity of the head injury does not consist of a critical factor on risk taking. Here patients regardless of the severity of their injury displayed very poor performance at the BART, pumping twice as less the balloons than healthy subjects (20 vs. 40 pumps). Furthermore, definition of the severity of head injury solely based on GCS scores (as in this study) may be insufficient. As Novack and colleagues [47] suggested “Defining the severity of injury based on a combination of factors (such as GCS score, duration of PTA, and signs of structural lesion on neuroimaging) is a commendable research goal”.

**Effects of frontal lobe status on risk taking in TBI patients.** Cicerone and colleagues [19] have proposed that the time course of improvement of cognitive functions postinjury in TBI varies depending on severity of focal and diffuse effects. Here, patients with a lesion in the frontal lobe of the right hemisphere (N = 14) and patients with no lesion in the frontal lobe of the right hemisphere (N = 16) performed differently during the second and third sets of balloons at the BART, but they did not differ during the first set of balloons. More specifically, patients with a lesion in the frontal lobe of the right hemisphere showed an increase risk taking as compared to patients with no lesion in the right frontal hemisphere, although they all started with a similar performance (i.e., for the first set). This was not observed in regards to the left hemisphere (18 patients with a lesion in the left frontal lobe and 12 patients with no lesion in the left frontal lobe). Moreover, we observed that performance of TBI patients with right hemisphere lesion differed even more from healthy controls than TBI patients with no right hemisphere lesion. As discussed above, healthy subjects usually show a learning curve throughout completion of the BART [30], [32]. Previous work reported that patients with frontal lesions display impaired risk taking and decision-making by engaging in more immediate, higher rewards (they bet more) than healthy subjects [52], [53]. There is however no clear consensus in regards to the hemispheric contribution on risk taking impairments in patients with frontal lesion. Some suggested that impaired risk taking and decision-making were linked to ventromedial lesions in the right hemisphere [43] – and our results support this –, but others proposed they were linked to ventromedial/orbitofrontal lesions regardless of the hemisphere [54], [55] or to the total frontal size and right nonventral frontal lesions [36], [44].

### Study Limitations

Several limitations should be considered when interpreting the present results. First, as discussed above, the sample size was small. Also, although the number of days between patient’s participation to the study and their injury was informative, that is indicating a curve of improved risk taking behaviors, a better design would have been to assess the same patients at different time points. We believe it was fortunate to evaluate TBI patients in their acute phase. However, we could assess them only once while they were inpatient and unfortunately it was not possible to follow them once they completed their inpatient care. More information on patients would also be important in future work to better understand risk taking in TBI. It would be important to further investigate the potential influence of lesion characteristics (e.g., size, precise location) using patient’s MRI on cognitive performance. In this study, we had access to the medical records that included the neurological report but not the scans. It would also be interesting to test the impact on risk taking in patients with concurrent conditions such as attention deficit hyperactivity disorder (ADHD), as ADHD has been associated with impaired risk taking [56]. Finally, inclusion of other tasks would also be informative to further interpretation of our findings. For instance, it would be interesting to assess functions known to be often impaired in TBI patients such as response speed [57], response inhibition [58], and attention [59]. These functions might have played a role in our results.

## Conclusion

In sum, this work shows that risk taking were impaired in patients with TBI during the acute phase. Patients displayed risk averse responses, as compared to healthy controls, to the simple BART task which has real world convergence [29–34]. Although risk taking seemed to improve naturally with time during the acute phase, it remained severely impaired. Also, within our TBI group, patients with frontal lesions in the right hemisphere took more risk at the BART than those with no right frontal lesion.

Although the BART experiment is rather simple compared to most cognitive tasks on risk taking that have been used in TBI (e.g., Iowa gambling task), patients here were severely impaired as compared to healthy controls (i.e., pumping twice as less the balloons). However, various processes still need to be integrated in order to earn money at this task. It is a goal-directed task involving reward seeking (i.e., to earn money) which demands initiating purposeful behavior (i.e., to pump or stop pumping the balloon) and anticipating consequences of their behavior (i.e., the more they pump, the greater money they earn, but so are the chances that the balloon explodes and then lose the money). It would be interesting to decipher which of these processes are impaired or intact in future work. As pinpointed by Donovan [60], assessment of cognitive functions following TBI is important for guiding rehabilitation. There is still no consensus on the optimal time window during which rehabilitation is more effective [61]: it has been suggested that it is best to intervene earlier [14] or later postinjury [62]. Longitudinal prospective studies assessing multiple cognitive skills should further elucidate potential relations between cognitive deficits and their natural improvement, as well as the impact of rehabilitation on these skills, to ultimately better guide programs.
